# Editorial: Clinical safety of natural products, an evidence-based approach

**DOI:** 10.3389/fphar.2022.960556

**Published:** 2022-08-16

**Authors:** Mojtaba Heydari, Abdur Rauf, Muthu Thiruvengadam, Xiao Chen, Mohammad Hashem Hashempur

**Affiliations:** ^1^ Persian Medicine Network (PMN), Universal Scientific Education and Research Network (USERN), Shiraz University of Medical Sciences, Shiraz, Iran.; ^2^ Department of Chemistry, University of Swabi, Swabi, Pakistan; ^3^ Department of Crop Science, College of Sanghuh Life Science, Konkuk University, Seoul, South Korea; ^4^ Department of Orthopedic Trauma, Shanghai Changhai Hospital, Second Military Medical University, Shanghai, China; ^5^ Research Center for Traditional Medicine and History of Medicine, Department of Persian Medicine, School of Medicine, Shiraz University of Medical Sciences, Shiraz, Iran

**Keywords:** natural products, herbal medicine, Persian medicine, safety, side effect, toxicity

There is increasing use of natural products including medicinal plants, phytopharmaceuticals, nutraceuticals, vitamins, and nutritional supplements for different health purposes worldwide (Mosavat et al., 2018). It is generally believed that these products are safe (Lynch and Berry, 2007). However, there is growing evidence of safety concerns associated with these natural products (Haq, 2004). Nevertheless, little is known about the adverse events associated with these products (Bent, 2008).

There are different concerns regarding the safety of natural products. The complexity in the nature of these formulations is one of the sources of concern (Capasso et al., 2003). Not only compound herbal formulations, but also simple herbal drugs have many biologically active ingredients which may have toxic effects. The interaction of the multiple herbs in a formulation and multiple ingredients in one herb with each other, make the safety evaluation more difficult than in chemical medicines. Besides the potential intrinsic toxicity of the natural products, extrinsic toxicity is another source of concern. For example, heavy metals are found at higher than standard levels in many herbal formulations on the market (Keshvari et al., 2021). Inaccurate identification of the used medicinal plants and their use in wrong clinical indications are other source of the safety issues. And the last point is the drug interactions between the natural products and chemical medicines taken by the patients (Ghosh et al., 2018).

Different methods have been popularly used to evaluate the safety of natural products. *In-vitro* and *in vivo* studies evaluating the toxicities of different cells and organs are among these methods. However, applying these data in clinical practice faces multiple limitations. Besides these traditional methods, different types of research including clinical safety studies and pharmacovigilance-based investigations can help us to have an evidence-based approach to the safety of these products (Raoufinejad et al., 2020). Information presented as associated adverse events of these products gathered from pharmacovigilance systems needs to be analyzed by scientific methods to determine the significance and potential causal relationship. The identification of adverse events associated with the use of natural products is challenging due to different reasons including impurities, batch-to-batch variability, misidentification and/or labeling, and different source of used production materials. There are also concerns about the suitable working of classic reporting systems in gathering reports on adverse events on unregulated medicines and supplements including so-called borderline products.

By collecting articles on this theme, we tried to develop information about the safety of natural products to enhance their proper use in the general population. In this regard, we focused on clinical, and epidemiologic studies in this field. Studies published in this research topic focused on the safety of medicinal use of multiple medicinal plants (*Tripterygium wilfordii, Entada abyssinica,* and *Brucea javanica*) and nutritional supplements (coffee and cinnamon) as natural products. We need to expand the scientific data on the clinical safety of natural products help us with evidence-based decision making on their proper use.
